# Endogenous and Exogenous Antioxidants as Agents Preventing the Negative Effects of Contrast Media (Contrast-Induced Nephropathy)

**DOI:** 10.3390/ph16081077

**Published:** 2023-07-28

**Authors:** Ina G. Panova, Alexander S. Tatikolov

**Affiliations:** 1 International Scientific and Practical Center of Tissue Proliferation, 29/14 Prechistenka Str., 119034 Moscow, Russia; pinag@mail.ru; 2 N.M. Emanuel Institute of Biochemical Physics, Russian Academy of Sciences, 4 Kosygin Str., 119334 Moscow, Russia

**Keywords:** contrast-induced nephropathy, contrast media, reactive oxygen species, oxidative stress, antioxidants, theranostics

## Abstract

The use of conventional contrast media for diagnostic purposes (in particular, Gd-containing and iodinated agents) causes a large number of complications, the most common of which is contrast-induced nephropathy. It has been shown that after exposure to contrast agents, oxidative stress often occurs in patients, especially in people suffering from various diseases. Antioxidants in the human body can diminish the pathological consequences of the use of contrast media by suppressing oxidative stress. This review considers the research studies on the role of antioxidants in preventing the negative consequences of the use of contrast agents in diagnostics (mainly contrast-induced nephropathy) and the clinical trials of different antioxidant drugs against contrast-induced nephropathy. Composite antioxidant/contrast systems as theranostic agents are also considered.

## 1. Introduction

The use of contrast media (CM) has become widespread in magnetic resonance imaging, computed tomography and radiography. The introduction of contrasting media (contrast agents) is carried out in cases where, during a standard scan, the physician cannot reliably determine the emerging pathologies. Special CM, which are usually administered intravenously to a patient, highlight pathological changes in organs and tissues in images and give specialists grounds to develop approaches for adopting a particular treatment. The technology provides physicians with the ability to obtain an accurate and detailed image of diseased organs, soft tissues and bones, allowing them to make the correct diagnosis. Among the many contrast agents presented in the literature [1,2,3,4,5], the most commonly used are iodine-containing CM for radiography and computer tomography and gadolinium (Gd)-containing CM for magnetic resonance imaging (MRI). These methods are considered effective for visual diagnostics and are used in the diagnosis of a wide range of diseases—pathologies of the brain and spinal cord, diseases of the spine and joints, some diseases of the abdominal cavity and pelvic area, heart, lungs, thyroid gland, etc. However, the introduction of CM causes side effects in various tissues of the body and entails serious complications [6,7,8,9,10].

Acute renal failure known as “contrast-induced acute kidney injury” (CI-AKI), also called “contrast-induced nephropathy” (CIN), is the main side effect of injection of CM and is still one of the most serious adverse complications, sometimes fatal [11,12]. This also results in an increase in its associated adverse reactions. All CM have direct cytotoxic effects on renal tubular epithelial and vascular endothelial cells and renal hemodynamics, leading to selective reduction in outer medullary blood flow, oxidative stress, apoptosis, immune/inflammation and epigenetic regulation [2,6,13,14,15,16,17,18]. Three main pathophysiological mechanisms of CIN have been proposed: direct tubular toxicity, intrarenal vasoconstriction and excessive production of reactive oxygen species (ROS), all leading to impaired renal function [12,19,20].

The exact mechanism of CIN is not fully elucidated. Infusion of contrast agents increase osmotic load and viscosity and increases hypoxemia of the renal medulla and renal free radical production through post-ischemic oxidative stress [13,21,22,23].

The excessive generation of ROS (oxidative stress) is believed to play a major role in the primary physiological insult and pathogenesis of CIN [19,21,24]; therefore, recently, a number of studies have focused on the potential role of antioxidants in the prevention of CIN. Antioxidants can decrease oxidative impairment in organisms directly by reacting with free radicals and indirectly by suppressing the activity and expression of free radical generating enzymes and stimulating the activity or expression of intracellular antioxidant enzymes [25].

Antioxidants are endogenous and exogenous, as part of the diet or a dietary supplement. Endogenous antioxidants are produced by the human body while exogenous antioxidants come from the human diet. Exogenous antioxidants can be of natural origin (fruits, vegetables, meat and fish products) or synthesized. Living organisms have developed a comprehensive set of endogenous antioxidant defenses to prevent the formation of excess free radicals or limit their damaging effects [26]. Antioxidants (exogenous and endogenous) in the human body can diminish the pathological consequences of the use of contrast media by suppressing oxidative stress [27]. But in patients (particularly elderly) with renal dysfunction and/or diabetes, the use of contrast media are risk factors and, in some cases, can be fatal [12].

This review considers, in particular, the research devoted to studying the role of antioxidants in preventing the negative consequences of the use of CM in diagnostics (mainly CIN). We did not aim to describe CM in more detail, since they are quite fully described in the literature [1,2,3,4,5,9,10,28]. Much attention in the review is given to clinical trials of drugs with antioxidant properties that can reduce or prevent CIN, as well as the induction of internal endogenous antioxidants against CIN. Composite antioxidant/contrast systems as theranostic agents are also considered.

## 2. Contrast Media

As mentioned above, there are two main types of CM used in clinical practice: iodine-containing (iodinated) and gadolinium-containing. The iodine-containing CM are divided into ionic and nonionic, as well as high-osmolar and low-osmolar (including iso-osmolar) with respect to the osmolarity of serum (about 290 mOsm/kg). Diatrizoate and metrizoate (ionic) pertain to high-osmolar iodinated CM (about 1500–2000 mOsm/kg); low-osmolar CM (600–1000 mOsm/kg) include ioxaglate and iothalamate (ionic) as well as iopamidol, iohexol, ioxilan, iodixanol and ioversol (nonionic) [9]. Hyperosmolality increases the intrinsic toxicity of iodinated CM. Along with oxidative stress, the administration of iodine-containing CM leads to other adverse processes, leading mainly to CIN [28]. The detailed scheme of pathophysiology of renal damage resulting from the administration of iodinated CM is presented in [16].

In gadolinium-containing CM, Gd^3+^ ion is complexed with different chelating agents. Such CM can be linear or cyclic, ionic or nonionic (e.g., Gd-DTPA—ionic, linear; Gd-DTPA-BMA—nonionic, linear; Gd-DOTA—ionic, cyclic; Gd-HP-DO3A—nonionic, cyclic) [1]. Nonionic complexes with low osmolality were developed to allow the use of higher doses [1]. The toxicity mechanisms (including CIN) of Gd-containing CM were considered in the review [29], in which elevation of ROS was noticed among other toxicity mechanisms. Cytotoxicity (nephrotoxicity) of Gd-containing CM is characterized by increased oxidative stress and mitochondrial dysfunction followed by cell death via apoptosis and, ultimately, necrosis [30].

Apart from Gd^3+^ chelates, commonly used as CM are manganese-based chelates and superparamagnetic iron oxide nanoparticles [1]. The use of nanoparticles in theranostics (as contrast/therapeutic agents) is considered in Section 5.

Recently, novel CM based on the detonation of nanodiamond (DND) particles with grafted paramagnetic metal cations have been developed [31,32,33,34]. Gd-grafted DND exhibit superior relaxivity properties, exceeding most of those known in the literature [31]. Saline suspensions of polyvinylpyrrolidone (PVP)-coated Gd-grafted DND used as MRI CM provide much higher signal intensities than the conventional CM Gd-DOTA, which increases its potency for a safer application in practice [32]. Mn-grafted DND particles have been prepared and studied [33,34]; their suspensions (with PVP-coated particles) are promising as MRI contrast agents [34].

Hence, novel DND particles with grafted paramagnetic metal cations hold much promise as CM in MRI and may be related to next-generation contrast agents for medical imaging.

## 3. Redox System

Normally, a balance between oxidative and reductive processes (redox system, redox homeostasis) is maintained in the organism. These processes ensure the integrity and basic functions of cells and maintain the viability of organs and tissues. The redox system regulates DNA synthesis, gene expression, enzyme activity, preservation and permeability of cell membranes and other processes [35,36,37]. An imbalance between the production and degradation of free radicals leads to oxidative stress. An excessive increase in ROS is a factor in cell and tissue damage as a result of destruction of cell membranes, proteins, nucleic acids, etc. [36,37,38,39,40]. The redox system maintains the equilibrium concentrations of ROS and antioxidants; it consists of two main arms of the balance: prooxidant and antioxidant.

The prooxidant system is represented by low molecular weight ROS. ROS include extremely reactive oxygen-containing free radicals and molecules, which are superoxide anion (O_2_^–•^), perhydroxyl radical (HO_2_^•^), hydroxyl radical (OH^•^), singlet oxygen (^1^O_2_), hydrogen peroxide (H_2_O_2_), nitric oxide (NO), hypochlorous acid (HOCl) and peroxynitrite (ONOO^–•^). They are generated as byproducts in the mitochondrial respiratory chain or synthesized by specialized enzymes (in particular, NADP oxidase, nitric oxide synthase). Reactive nitrogen species (RNS) are formed from NO via reaction with O_2_^–•^ to form ONOO^–•^ [25,37,40,41,42].

The destructive activity of ROS consists of the oxidation of lipids, proteins and DNA [30]. Oxidized forms of proteins, when accumulated excessively, can aggregate and cause additional endoplasmic stress and unfolded protein responses [43].

Another important factor in the pathogenesis of CIN is the induction of endothelial dysfunction and changes in renal microcirculation. ROS play a key role in these phenomena through the production of vasoconstrictors [44]. Increased ROS can reduce the bioavailability of nitrogen oxide (NO) [45].

On the other hand, ROS, while being unstable, affect the activity of a number of enzymes (proteinase, phosphatase, phospholipase, etc.) and modulate the expression of transcription factors, thereby providing global stable changes in gene activity and cell metabolism. For example, ROS (in particular, hydrogen peroxide), can reversibly oxidize critical, redox-sensitive cysteine residues on target proteins. These oxidative post-translational modifications can control the biological activity of numerous enzymes and transcription factors, as well as their cellular localization or interactions with binding partners [46]. The molecular pathways of ROS regulatory action are still insufficiently studied, but their leading role in various physiological processes and signaling pathways is actively discussed [46].

The antioxidant system includes low molecular weight reducing agents (vitamin C, glutathione, taurine, uric acid, cysteine, beta-carotene, etc.) and enzymatic systems that neutralize ROS (superoxide dismutase, catalase, glutathione peroxidase, peroxiredoxins, etc.) or reduce oxidized proteins and lipids (e.g., glutathione enzyme system) [47]. Endogenous and exogenous antioxidants can work synergistically to maintain or establish redox balance. ROS and antioxidants interacting with each other are considered functionally related redox-active molecules; they are key components of redox processes in organisms [48].

Due to the fact that, when CM is administered, oxidative processes increase dramatically and lead to tissue impairment (patients with kidney failure, diabetes and obesity are especially susceptible), there is an urgent problem in preventing the occurrence of CIN by using antioxidants for the neutralization of ROS before administration or during administration of CM. It is also important to choose suitable antioxidant agents for the treatment of CIN.

## 4. Antioxidants vs. Contrast-Induced Nephropathy

### 4.1. N-Acetyl-L-Cystein/Glutathione

**Figure 1:**
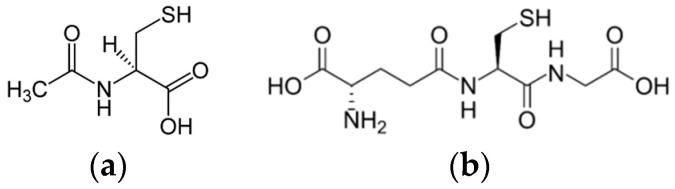
Structures of (**a**) N-acetyl-L-cystein (NAC) and (**b**) glutathione (GSH).

N-Acetyl-L-cystein (NAC, Figure 1a) is a thiol-containing exogenous antioxidant that has been applied clinically for about 70 years, mainly for mucolytic therapy in respiratory diseases [49]. In an organism, NAC deacetylates with conversion to cysteine, being a precursor for the endogenous cellular antioxidant—glutathione (GSH) [50]. Administration of NAC (via orally or intravenously) provides cysteine as a substrate for replenishing glutathione stores in cells [51].

Due to its antioxidant ability (possibly indirect [52]), NAC is widely used as a remedy for CIN with good results (see, e.g., [53,54,55,56,57,58], meta-analysis [59], with intravenous administration [60], meta-analysis at high doses of CM [61]). At the same time, there are many publications that do not confirm the efficacy of NAC in diminishing CIN (see, e.g., [62,63,64,65,66,67], meta-analysis for oral NAC administration [68], meta-analysis for intravenous NAC administration [69]). This could be in part due to some side effects of NAC, in particular, its pro-oxidant properties [70]. The meta-analysis of the 61 randomized clinical trials made in [71] showed that the incidence of CIN in the NAC group of patients compared with that in the control group revealed a statistically significant difference. In patients undergoing coronary angiography, the incidence of CIN in the NAC and control groups was also statistically different. For patients undergoing computed tomography, the difference between the corresponding incidences was about twofold, while no difference was observed for patients undergoing peripheral angiography [71]. However, the recently completed largest meta-analysis of 101 randomized control trials has shown that NAC has practically no effect on CIN prevention [72]. Keeping in mind the data of meta-analyses on the whole, NAC should be thoroughly examined and recommended mainly as an additional therapy for CIN [73]. Note that the recent publication focuses on the antioxidant and anti-inflammatory activity of NAC, which can help not only in CIN but also in chronic kidney disease [74].

**Figure 2:**
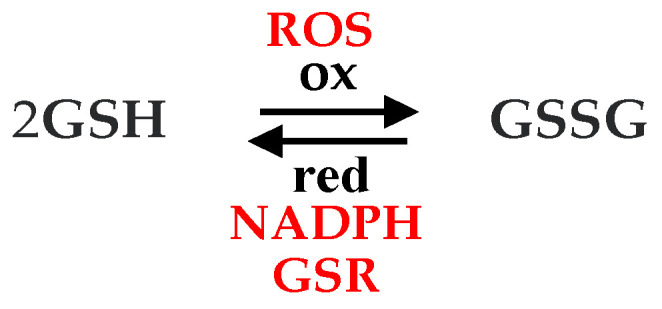
Scheme of redox equilibrium involving GSH.

Glutathione (GSH, Figure 1b), a cellular antioxidant, is a sulfur-containing tripeptide synthesized from cysteine, glutamate and glycine mainly within the liver [75]. It performs various functions in an organism. In particular, it controls gene expression and apoptosis, detoxifies drug metabolites, affects cell responsiveness to redox changes brought on by ROS and participates in the transmembrane transport of organic solutes. GSH is a desirable target for a closer approach to the prevention and treatment of numerous illnesses of concern to physicians [75]. It participates in a redox equilibrium with its disulfide (oxidized) form (GSSG). The antioxidant action of GSH consists of oxidation of GSH to GSSG by ROS, which leads to their inactivation. GSSG formed is reduced back to GSH by NADPH catalyzed by glutathione reductase (GSR) (Figure 2).

GSH directly scavenges diverse oxidants in cells: superoxide anion, hydroxyl radical and carbon radicals; it catalytically detoxifies hydroperoxides, peroxynitrites and lipid peroxides [76]. It also protects cells from oxidants by recycling of other antioxidants—vitamins C and E [77]. Therefore, it was reasonable to test GSH against oxidative stress caused by CIN. However, the studies on the influence of reduced GSH (together with hydration) on preventing CIN in patients gave no distinct results [78,79,80]. The lack of GSH effects on CIN in patients found in these studies could be due to insufficiently small sample sizes; therefore, larger prospective randomized GSH trials are required.

The recent publication suggests the use of GSH sodium salt with another antioxidant—ascorbic acid, to suppress CI-AKI in patients with contrast-associated ST-elevation myocardial infarction (STEMI) [81]. It seems to be promising and should be a matter of further investigations.

### 4.2. L-Ascorbic Acid (Vitamin C)

**Figure 3:**
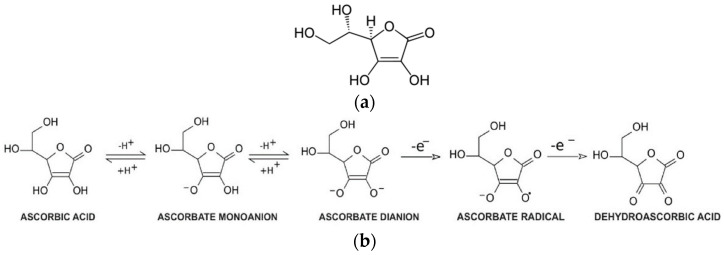
(**a**) Structure and (**b**) acid-base and redox transformations of ascorbic acid.

An important compound with antioxidant properties is vitamin C (L-ascorbic acid, Figure 3a). It is a water-soluble organic compound present in living organisms and in food. Vitamin C at physiological pH mainly exists in the form of ascorbate anions, which can be oxidized to dehydroascorbic acid (Figure 3b). In humans, vitamin C is not synthesized, but is supplied exclusively with food [82,83,84].

In human cells, L-ascorbic acid is transported via sodium-dependent vitamin C transporters (SVCT) SVCT1 and SVCT2, whereas the oxidized form of vitamin C, dehydro-L-ascorbic acid (DHAA), via glucose transporter (GLUT). The low affinity transporter SVCT1 is mainly responsible for the uptake of ascorbate by intestinal epithelial cells. The high affinity transporter SVCT2 delivers ascorbate to body tissues. After being transported across the cytoplasmic membrane, vitamin C accumulates in cells. After entering a cell, DHAA is rapidly reduced to L-ascorbic acid [85,86,87].

Vitamin C at very low concentrations and in the presence of trace amounts of transition metals (iron, copper) acts as a pro-oxidant, promoting the process of lipid peroxidation catalyzed by metals. However, at higher contents, in particular, at physiological concentrations (about 40–80 μM) in the blood plasma of healthy humans, vitamin C functions as an antioxidant [83]. It can reduce the oxidized radical form of vitamin E (α-tocopheroxyl radicals, α-TO^•^), thus regenerating and prolonging the life cycle of this antioxidant in the lipid phase and facilitating the removal of radicals from the lipid to the aqueous phase [88]. Ascorbic acid synergistically interacts with vitamin E and thus protects low density lipoproteins from oxidative damage by peroxyl radicals. Vitamin C is extremely important for maintaining normal vitamin E levels and inhibition of lipid oxidation [89].

Vitamin C can modulate gene expression and is involved in cell differentiation processes [87].

Ascorbic acid, due to its antioxidant properties and ability to stimulate collagen synthesis, inhibits angiogenesis and reduces the permeability of blood vessels [90,91]. Ascorbic acid has also been reported to cause vasodilation. The vasodilatory effects of ascorbic acid were observed, in particular, in patients with non-insulin-dependent diabetes mellitus [92] and in essential hypertensive patients [93]. These properties of ascorbic acid may be useful in preventing CIN risk. Indeed, there is evidence (meta-analyses) that ascorbic acid reduces the risk of developing CIN [94,95].

It has been shown that vitamin C (given at doses of 1–3 g in combination with hydration prior to coronary angiogram) can have a significant effect in the prevention of CIN among high-risk patients and can be recommended to prevent the development of CIN in patients with renal insufficiency. It is safe, inexpensive and readily available [95,96,97]. It is also suggested (in combination with GSH sodium salt) for suppressing CIN in patients with ST-elevation myocardial infarction (STEMI) [81]. Ascorbic acid has a dose-dependent protective effect on renal cells, preventing contrast-induced apoptosis [98]. However, there are some conflicting data on the effectiveness of ascorbic acid in reducing the development of CIN. A number of studies have shown the absence of a nephroprotective effect of ascorbic acid upon administration of contrast agents [99,100,101,102,103]. No effects of ascorbic acid and NAC on CIN in critical care patients were found in [104]. Such contradictions require further research.

### 4.3. Vitamin E (Tocopherols, Tocotrienols)

Vitamin E is the name of eight natural compounds: α, β, γ and δ derivatives of tocopherol and tocotrienol, which are exogenous lipophilic antioxidants.

**Figure 4:**
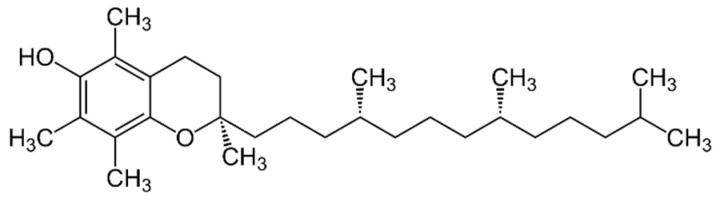
Structure of α-tocopherol.

α-Tocopherol (Figure 4) is a more active antioxidant [105], while γ-tocopherol has higher anti-inflammatory properties [106]. α-Tocopherol operates in the glutathione peroxidase pathway [107] and protects cell membranes from oxidation by reaction with lipid radicals produced in the lipid peroxidation chain reaction [108]. The oxidized α-tocopheroxyl radicals formed in this process may be recycled back to the active form via reduction by other antioxidants (ascorbate, retinol or ubiquinol) [109].

Different forms of vitamin E were proposed to prevent several diseases, especially arteriosclerotic heart disease and cancer [110,111], primarily due to their antioxidant and anti-inflammatory properties [106,108,112]. Due to its profound antioxidant properties, α-tocopherol was often used for prevention or attenuation of CIN. Some meta-analyses of the studies of its effect on CIN have been performed. In particular, [113] concluded that α-tocopherol administration (oral or multiple) leads to a reduction in CIN incidence and should be considered a simple and inexpensive remedy for CIN prevention. In [114], a meta-analysis showed that vitamin E together with hydration significantly reduced the risk of CIN in patients with renal impairment. However, no effect of α-tocopherol for prevention of CIN was found in [115]. No effect of vitamin E on CIN was found in [116] either.

### 4.4. Bilirubin

**Figure 5:**
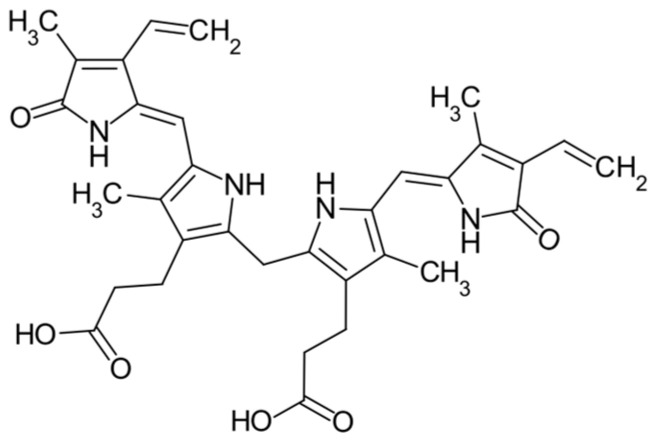
Structure of bilirubin.

Bilirubin is a bile pigment, i.e., a component of bile in humans and animals, possessing antioxidant properties [117,118] (Figure 5).

Bilirubin has been demonstrated to offer renal protection in several models of acute kidney injury [119,120]. Low serum bilirubin concentrations predict the development of chronic kidney disease and diabetic nephropathy, whereas higher levels of serum bilirubin are associated with a decreased risk of diabetic nephropathy [121,122,123].

A number of works appeared on the relationship between CIN and serum bilirubin as an endogenous antioxidant molecule. Some studies showed that patients with CIN had a lower level of total bilirubin compared to control patients [124]. These studies suggested that a general decrease in bilirubin was associated with the development of CIN in patients after the administration of radiocontrast agents. Indeed, patients with lower total serum bilirubin levels had a higher incidence of CIN after the use of CM. Higher serum bilirubin concentrations were associated with a lower risk of CIN and fewer cardiovascular complications [124,125]. As a result of these observations, the authors concluded that further studies are needed to identify the exact mechanisms of bilirubin involvement in the prevention of CIN in clinical practice [125]. The development of interventions that increase serum bilirubin levels may be a potential target for the prevention and lowering of CIN [124].

Analysis of the known properties of bilirubin can lead to an understanding of the importance of bilirubin in protecting against CIN.

**Figure 6:**
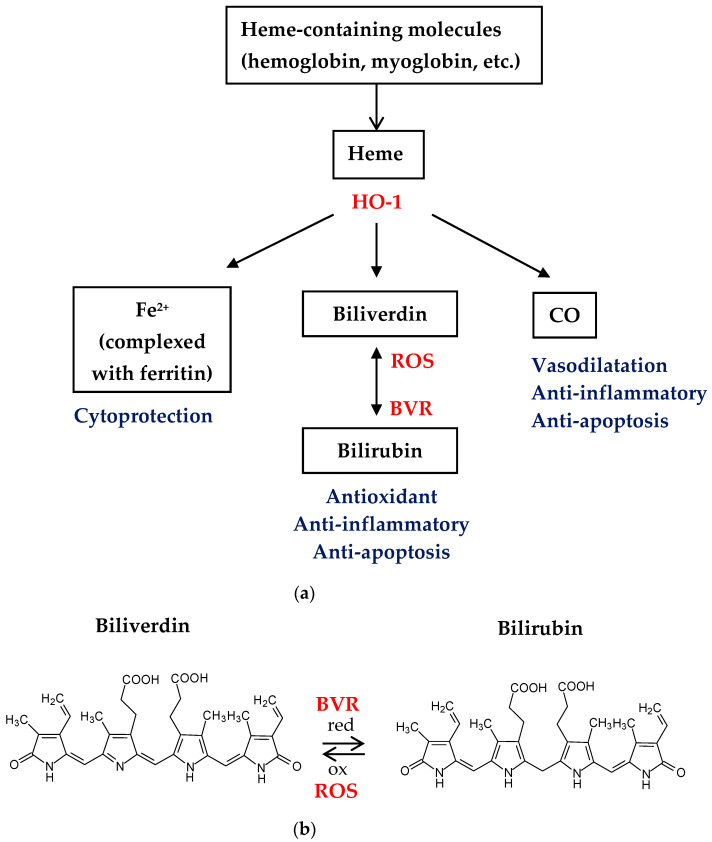
(**a**) Scheme of bilirubin production and (**b**) biliverdin–bilirubin redox equilibrium.

Bilirubin is the end product of the metabolism of heme-containing protein molecules—hemoglobin, myoglobin and some heme-containing enzymes (cytochrome, catalase, peroxidase). The main supplier of bilirubin is hemoglobin of erythrocytes. Erythrocytes undergo destruction intracellularly (in macrophages) [117,118]. Cleavage of heme to water-soluble biliverdin occurs through the involvement of the heme oxygenase-1 (HO-1) enzyme. Biliverdin is then reduced by biliverdin reductase (BVR) to water-insoluble bilirubin (Figure 6).

Normally, most of circulating bilirubin is unconjugated and circulates in plasma bound to albumin. Unconjugated bilirubin is a powerful endogenous serum antioxidant and is one of the important protective agents against oxidative stress. Lipid peroxidation by free oxygen radicals is the most important cause of cell membrane damage and cell destruction. Bilirubin, being a lipophilic molecule, passes through membranes, is incorporated into cells and organs and protects cell membranes from lipid peroxidation [126,127,128]. The antioxidant activity of bilirubin in the vascular endothelium may be a dynamic factor in endothelial function and determines the physiological redox homeostasis of the vascular endothelium [129].

It has also been shown that, along with antioxidant ability, bilirubin has other important biological properties, which include anti-inflammatory, immunomodulatory, cytoprotective and neuroprotective activities [130,131,132,133,134].

The scheme of bilirubin production from heme-containing molecules is shown in Figure 6a. Heme-containing molecules (hemoglobin, myoglobin, etc.) under the influence of heme oxygenase-1 (HO-1) are converted into biliverdin (in parallel with the formation of Fe^2+^ and CO). Biliverdin reductase (BVR) reduces biliverdin to bilirubin—a potent lipophilic ROS scavenger, which effectively protects cells from various oxidative stresses and regulates the expression of HO-1, another potent antioxidant. In this scheme, the two cycles of the ROS scavenging system are interplayed. The first cycle is the redox conversion of biliverdin to bilirubin by BVR with the reverse conversion of bilirubin to biliverdin probably by lipophilic oxidants (Figure 6b). The second cycle is the induction of HO-1 by BVR and the degradation of heme to biliverdin, which is again metabolized to bilirubin by BVR [135]. HO-1 is an inducible enzyme with anti-apoptotic and antioxidant properties. Its antioxidant ability is due to participation in the production of the biliverdin–bilirubin system, which scavenges free radicals, and in the decomposition of hydrogen peroxide in the biliverdin-to-bilirubin conversion process. In the course of bilirubin production, carbon monoxide is also formed which possesses anti-inflammatory properties [136]. The products of heme metabolism (Fe^2+^, biliverdin, bilirubin, CO), the enzymes HO-1 and BVR and the pathways for converting biliverdin to bilirubin and vice versa represent a powerful antioxidant and anti-inflammatory defense system [137].

Given the remarkable antioxidant and anti-inflammatory properties of bilirubin and the role of inflammation and oxidative stress in the pathogenesis of CIN and atherosclerosis, it would be interesting to initiate a clinical intervention that could raise serum bilirubin levels, possibly by inducing HO-1, as a potential strategy to prevent CIN [138]. Indeed, bilirubin levels are shown to be associated with CIN [124,125,139]. Studies on rats demonstrated the efficacy of HO-1 inducer (hemin) for CIN mitigation [140]. Along with hemin and heme-containing molecules, HO-1 can be induced by various other biomolecules [141,142]. Hence, the development of interferences stimulating bilirubin levels by induction of HO-1 may be a potential target to reduce CIN and future adverse outcomes in patients with coronary intervention. Note that, along with the beneficial effects of HO-1 induction, some studies indicate that the increased HO-1 expression may lead to the development of several diseases, such as neurodegeneration and carcinogenesis [143]. Note also that high levels of total serum bilirubin are toxic (hyperbilirubinemia) [144]. Therefore, more thorough studies of HO-1 induction consequences in patients with CIN, as well as the appropriate experimental studies involving animal models, are needed.

### 4.5. Melatonin

**Figure 7:**
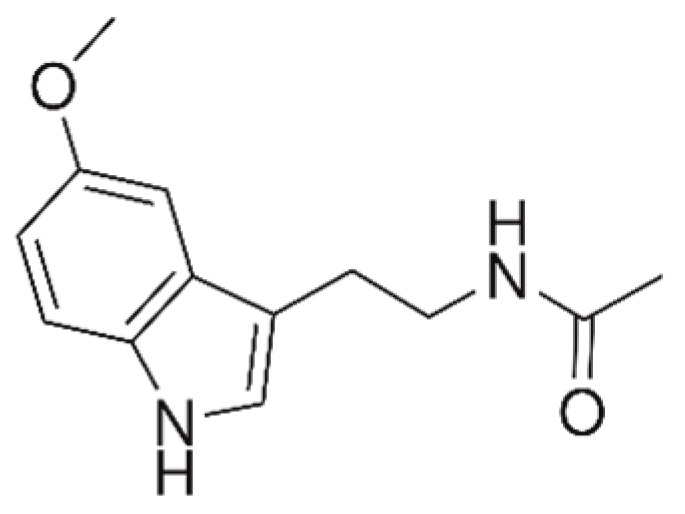
Structure of melatonin.

Melatonin (5-methoxy-N-acetyltryptamine) is an endogenous neurohormone (Figure 7). The production of melatonin is dependent on the light/dark cycle. It is a secretory product of the pineal gland and epithelial layer of the entire gastrointestinal tract; it has free radical-scavenging and strong antioxidant properties.

It protects tissues against oxidative damage induced by various free radical-generating agents and processes [145]. Melatonin also takes part not only in antioxidative, but in anti-inflammatory, antiapoptotic and immune processes [146,147,148,149]. Melatonin also stimulates glutathione peroxidase activity which metabolizes the precursor of the hydroxyl radical, i.e., hydrogen peroxide, to water [150].

Apart from its lipophilic properties, melatonin has some hydrophilic properties [151]. It can diffuse easily into subcellular compartments, thereby providing on-site protection against free radical-mediated damage and is a protective agent to cells and biomolecules [152].

The melatonin molecule interacts with free radicals to form metabolites that are also effective as free radical scavengers. Moreover, the antioxidant effects of melatonin are probably also based on the stimulatory effect on the formation of superoxide dismutase (SOD), GSH peroxidase, GSH reductase, glucose-6-phosphate dehydrogenase and the inhibitory effect on the expression of nitric oxide synthase (NOS) [150,152].

Due to the unique properties of melatonin as a powerful endogenous scavenger of free radicals, as immunomodulator and antioxidant, with anti-inflammatory and anti-apoptotic properties as well as being non-toxic, melatonin is apparently capable of providing a universal protective role in organisms and is a promising remedy for ameliorating CIN. From this viewpoint, melatonin has begun to be studied as a potential remedial agent for CIN. In experiments on animals (rabbits, rats), melatonin, in combination with hydration, has been shown to have a protective effect against CIN after exposure to CM [152,153,154]. It was shown in a mice model that melatonin mitigates CIN by the activation of Sirtuin-3 (Sirt3), which attenuates tubular epithelial cell apoptosis, oxidative stress and mitochondrial dysfunction [146]. Melatonin was shown (in rats) to attenuate oxidative stress, NLRP3 inflammasome and apoptosis induced by CIN [155]. Recently, it was concluded that supplementation with melatonin can be helpful in almost every type of kidney injury because inflammation, apoptosis and oxidative stress occur regardless of the mechanism [149]. Thus, the application of melatonin is very promising for the attenuation of CIN. However, for the successful use of melatonin as a therapeutic agent, the choice of adequate dose is extremely important. It can no longer be an a priori standard and should depend on the time of day and the initial endocrine status of the patient. In this regard, a continuation of experimental studies on the role of preventive melatonin treatment on animal models and their extension to clinical trials is needed.

### 4.6. L-Carnitine

**Figure 8:**
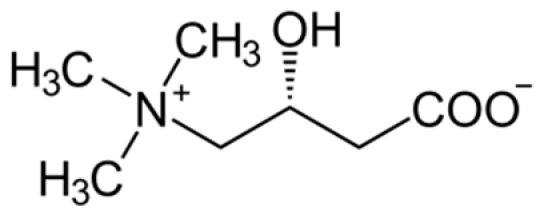
Structure of L-carnitine.

L-Carnitine (β-hydroxy-γ-trimethylammonium-n-butyric acid, see Figure 8) plays an important role in supporting the metabolic activities of organisms.

In support of energy metabolism, carnitine transports long-chain fatty acids into mitochondria to be oxidized for free energy production and also participates in removing the products of metabolism from cells [156,157,158]. It is biosynthesized mainly in the liver, kidney and brain from the essential amino acids lysine and methionine [159]. It has been shown that L-carnitine inhibits free radical generation, preventing the impairment of fatty acid oxidation in mitochondria and protecting tissues from damage by repairing oxidized membrane lipids [160,161]. Its antioxidant activity is comparable to that of α-tocopherol [161].

Many experimental studies have shown that L-carnitine reduces drug-induced nephropathy via several mechanisms, such as anti-inflammatory effects, antioxidative activity by the inhibition of ROS generation and lipid peroxidation, inhibition of matrix remodeling and apoptosis [162,163]. Recent studies showed the efficacy of L-carnitine against CIN in patients undergoing percutaneous coronary intervention [163,164,165]. Hence, L-carnitine can be considered a preventive treatment against CM agents. However, to support this statement more strongly, further comprehensive well-designed human studies are needed.

### 4.7. Statins

Statins, also known as 3-hydroxy-3-methylglutarylcoenzyme A reductase inhibitors, are a potent class of inhibitors of cholesterol biosynthesis and therapy agents for prevention of cardiovascular diseases. Their multiple effects, such as anti-inflammatory, antioxidant, antiproliferative and immunomodulatory effects, as well as plaque stability, normalization of sympathetic outflow and prevention of platelet aggregation are due to reduction in circulating isoprenoids and hence inactivation of signaling protein diseases [166,167,168]. The antioxidative properties of statins play a role in prevention of atherosclerosis [169]. Statins are also being widely used for the prevention of CIN (see, e.g., [170,171,172,173]).

A number of meta-analyses of data available on statin application have been performed [174,175,176,177] which show the efficacy of using statins against CIN. The antioxidant role of statins upon attenuation of CIN, which includes modulation of oxidative stress and nitric oxide, has been demonstrated on animals [178,179]. However, it was mentioned in the review [180] that, regarding the efficacy of statins on patients for preventing CIN, there are also controversial results, most likely due to the marked heterogeneity of patient characteristics, dosage and administration type of statins, definition of CIN and different statistical analyses.

### 4.8. Probucol

**Figure 9:**
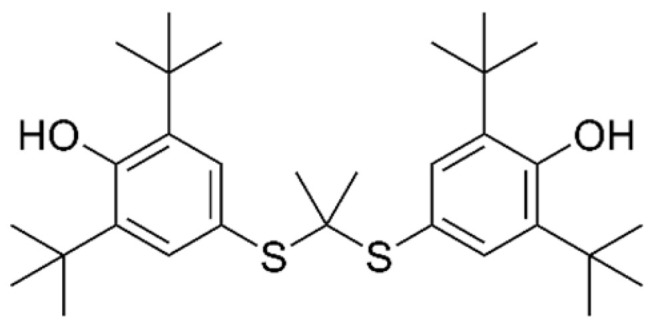
Structure of probucol.

Probucol is an antilipidemic drug initially developed for the treatment of coronary artery disease (Figure 9).

It is a lipid-lowering drug with strong anti-lipid peroxidation and anti-inflammatory properties, i.e., a powerful antioxidant (bisphenol) which inhibits the oxidation of cholesterol in low-density lipoproteins. It slows the formation of foam cells, which form atherosclerotic plaques. It reduces endogenous nitric oxide synthase inhibitor concentration, improving the renovascular endothelial function. Additionally, it increases the synthesis of prostacyclins, suppresses the expression of different adhesion molecules and helps proliferation of endothelial cells, preventing their apoptosis due to oxidative injury [181,182]. Probucol is often used in clinical practice to prevent and treat atherosclerosis and diabetic nephropathy. In addition, some studies have shown that probucol can be used as a prophylactic in developing CI-AKI. [183] The antioxidative stress and anti-inflammatory effects of probucol may help to prevent the occurrence of CI-AKI in patients older than 18 years with coronary heart disease undergoing coronary percutaneous intervention [181]. Some meta-analyses were performed on the efficacy of probucol in the incidence of CIN. In [184], it was shown that probucol did not reduce the incidence of CIN. However, another meta-analyses [183,185] showed that probucol with hydration decreased CIN incidence when compared to hydration in patients undergoing coronary angiography or percutaneous coronary intervention. It was concluded that more high-quality, large-sample, multicenter randomized trials are necessary to support this finding.

### 4.9. MESNA

**Figure 10:**
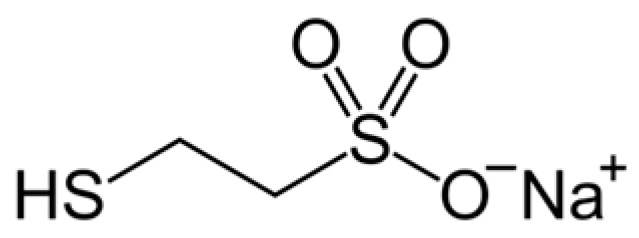
Structure of MESNA.

MESNA (2-mercaptoethane sulfonate, Figure 10) is a medication used to reduce the incidence of hemorrhagic cystitis and hematuria when a patient receives ifosfamide or cyclophosphamide for cancer chemotherapy. It is a small molecule containing an antioxidant SH group that has the potential to scavenge ROS and prevent glutathione depletion through thiol group formation [186,187,188,189]. Experiments in vitro have shown that the antioxidant MESNA reduces tissue damage caused by free oxygen radicals in the proximal kidney tubules [190]. Studies with animal models of acute renal failure have shown that MESNA pretreatment scavenges ROS and diminishes renal injury [191]. Intravenous administration of MESNA by patients before or after administration of a contrast agent had a protective effect on the kidneys. The studies suggested that MESNA is able to prevent CIN [190,192]. Further investigations on MESNA application for preventing CIN are necessary.

### 4.10. Resveratrol

**Figure 11:**
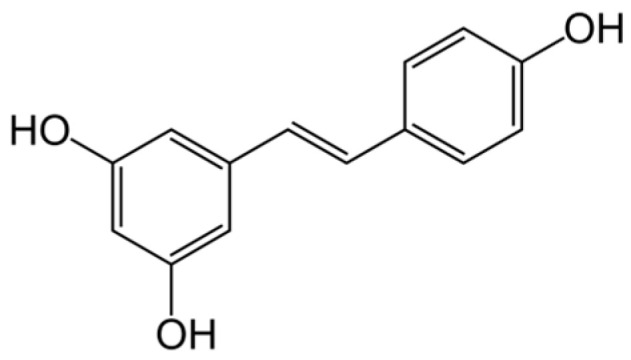
Structure of resveratrol.

Resveratrol (3,5,4′-trihydroxy-*trans*-stilbene) is a natural polyphenol, possessing antioxidant properties (Figure 11). It occurs in berries, such as redcurrants, cranberries, lingonberries, etc. [193].

Resveratrol possesses a wide range of biological properties, among them antioxidant, cardioprotective, neuroprotective, anti-inflammatory and anticancer activities [194]. Resveratrol activity against CIN was studied on a number of model systems. It was shown on mice that resveratrol attenuated CIN by modulating renal oxidative stress and apoptosis through activation of SIRT1-PGC-1α- FoxO1 signaling [195]; it was found on rabbits that resveratrol reduced renal hypoxia, mitochondrial dysfunction and renal tubular cell apoptosis by activating SIRT1–PGC–1α–HIF-1α signaling pathways in CIN with diabetic nephropathy [196]. Using a human renal proximal tubule epithelial cell line (HK-2 cells), it was shown that resveratrol may prove to be an effective add-on therapy for the prevention of CIN [197,198]. A rat CIN model showed that resveratrol treatment attenuated both injury processes and apoptosis and inhibited the inflammasome pathway in CIN [199]. The effects of resveratrol and lycopene (carotenoid) on CIN in rabbits were studied. It was shown that both resveratrol and lycopene ameliorated CIN by modulating oxidant/antioxidant balance in blood/renal tissue and by inhibiting vasoconstriction/blood cytotoxicity [200]. This medication needs further study.

### 4.11. Carotenoids

Carotenoids are a class of naturally occurring yellow, orange and red pigments synthesized by plants, algae and photosynthetic bacteria. Carotenoids from the diet are stored in the fatty tissues of animals [201]. Carotenoids possess antioxidant properties: they are efficient antioxidants scavenging singlet oxygen and peroxyl radicals. In the human organism, carotenoids are part of the antioxidant defense system [202].

**Figure 12:**
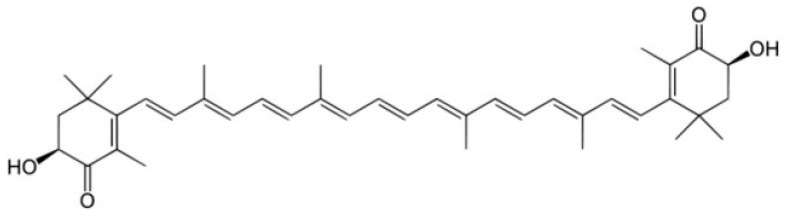
Structure of astaxanthin.

Astaxanthin is a xanthophyll carotenoid (Figure 12) with potent antioxidant and anti-apoptosis effects demonstrated in both experimental and human studies.

The antioxidant activity of astaxanthin toward peroxyl radicals in liposomes was shown to be higher than that of α-tocopherol, β-carotene, lutein and lycopene [203]. Many studies have proven that astaxanthin has a preventive effect on various kidney diseases [204,205,206]. The review in [207] summarizes the available evidence demonstrating that astaxanthin may be of therapeutic value in CIN.

**Figure 13:**
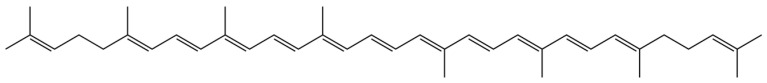
Structure of lycopene.

Lycopene is a carotenoid with antioxidant properties (Figure 13) found in tomato and other red fruits and vegetables [208].

Lycopene was shown to be protective in oxidation of lipids, proteins and DNA in vivo and to exhibit protective effects against different nephrotoxic agents [209,210]. Lycopene exhibited anti-inflammatory, antiautophagic and antiapoptotic properties in an experimental model of CIN in rats [211]. The effects of lycopene and resveratrol on CIN in rabbits were studied (see above). It was found that both compounds attenuated CIN by modulating oxidant/antioxidant balance in blood/renal tissue and by inhibiting vasoconstriction/blood cytotoxicity [200].

### 4.12. Plant Antioxidants

Plant extracts with antioxidant properties have been widely applied as remedies. Some of them are used for the prevention, relief and treatment of CIN (mostly in animal models). They are thoroughly considered in the recent reviews [212,213], so below we present only some representative examples.

#### 4.12.1. Green Tea Extract

Green tea (Camellia sinensis) is a popular herbal remedy worldwide. Polyphenols in green tea have attracted much attention as potential compounds for the maintenance of human health due to their biological activities and low toxicities. In recent years, the remedial effects of green tea on injury caused by oxidative stress have been the focus of research [214,215,216,217]. Green tea has antioxidant and anti-inflammatory properties which are due to its polyphenolic compounds and flavonols, such as catechins, gallic acid (epigallocatechin gallate, epicatechin gallate) and phenolic acids [218]. Studies on Wistar rats showed the efficacy of green tea extract against CIN [219,220]. The data presented evidence that green tea has sufficient antioxidant potential to protect tubular renal cells from CIN. In this regard, extensive experimental and clinical trials are necessary to study this remedy in more detail.

#### 4.12.2. Grape Seed Proanthocyanidin Extract

Grape seed proanthocyanidin extract (GSPE) is derived from grape seeds. In vivo and in vitro studies have shown that GSPE has a stronger antioxidant effect than vitamins C and E [221]. GSPE is a combination of biologically active polyphenolic flavonoids, including oligomeric proanthocyanidin, which are found in vegetables, fruits and various flowers [222]. In addition to its lipid peroxidation, thrombocyte aggregation and capillary permeability-reducing effects, GSPE also has antibacterial, antiviral and anti-inflammatory properties. GSPE exhibits these effects by modulating various enzymes, including cyclooxygenase and lipoxygenase [222,223]. It has been shown on rats that GSPE effectively prevents CIN [224]. In preventing CIN, GSPE was shown to be superior to NAC, which may be due to a decrease in calpain 1 levels [225]. This improvement was associated with the decrease in oxidative damage and apoptosis. The vasodilator, antiallergic, cardioprotective and immunomodulator characteristics of GSPE have been shown in various experimental studies in addition to its ROS scavenging and antioxidant features [222,223,224]. Further comprehensive and detailed experimental and clinical studies are needed to investigate the preventive effects of GSPE on CIN.

#### 4.12.3. Curcumin

**Figure 14:**
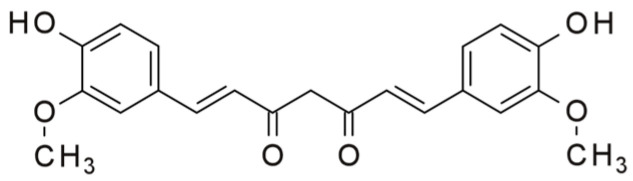
Structure of curcumin.

Curcumin is a compound produced by plants of the Curcuma longa species. It is the principal curcuminoid of turmeric and a food additive. Chemically, curcumin is a diarylheptanoid, belonging to the group of curcuminoids—phenolic pigments responsible for the yellow color of turmeric (Figure 14).

The properties of curcumin include antioxidant, anti-inflammatory, antiviral, antifungal and protective effects against chemical toxicities. Curcumin directly exhibited the antioxidant ability by free radical scavenging and indirectly by inducing an antioxidant response [226]. The nephroprotective effect of curcumin was evaluated in experimental models including nephrotoxic drugs, chronic renal failure, diabetic nephropathy and ischemic nephrotoxicity [227].

It has been found that curcumin can attenuate CIN by upregulating HO-1 expression (shown in rats) [228]. Furthermore, it increases GSH, superoxide dismutase, catalase and GSH peroxidase levels [229]. Since curcumin is safe and does not have major toxicity [230], it has the potential to be used against CIN.

### 4.13. Novel Antioxidant—Xylose–Pyrogallol Conjugate

Xylose–pyrogallol conjugate (XP; chemical name 1-Deoxy-1,1-bis(2,3,4-trihydroxyphenyl)-D-xylitol) was synthesized as a new antioxidant exerting a protective effect on CIN through the regulation of mitochondrial function, oxidative stress and apoptosis [231]. XP was studied on a CIN model on rats. The study indicated that XP played a nephroprotective role probably via antiapoptotic and antioxidant mechanisms. Furthermore, XP may regulate the mitochondrial pathway. It has been concluded that XP as an efficient antioxidant may have the potential to prevent CIN [231].

### 4.14. Summary

Qualitative results of clinical trials and research studies of antioxidant remedies used for prevention and attenuation of CIN are summarized in Table 1.

## 5. Hybrid Contrast/Antioxidant Media as Theranostic Agents

A new approach for obtaining antioxidants for the prevention of CIN is to develop hybrid contrast/antioxidant complex systems possessing theranostic properties, that is, being both CM (for diagnostic purposes) and antioxidants (for therapy). Such systems often have improved contrast properties in comparison with conventional CM (e.g., lower toxicity) and, along with suppressing CIN, have a rather wide range of therapeutic abilities (e.g., anti-inflammatory, anti-cancer activities). Some such systems are presented below.

### 5.1. Gd Complex/Rosmarinic Acid Conjugate

Rosmarinic acid (RosA) is a polyphenol known for its antioxidant and anti-inflammatory properties [232]. To join in one molecule antioxidant and contrast properties, a conjugate containing Gd–DOTA complex and RosA was synthesized (Gd–DOTA–RosA) and studied on mice [233]. This conjugate combined an MRI agent (Gd–DOTA complex) with an antioxidant and anti-inflammatory drug (RosA) and enabled the diagnosis of inflamed tissues via MRI. Its relaxivity is higher than that of Gd-BT-DO3A, and its kinetic stability is similar to that of structurally related Gd chelates. The antioxidant and anti-inflammatory activities of this agent exploited the radical-scavenging effects, the inhibition of COX-2 production and suppression of the proinflammatory cytokine TNF-α [233]. This conjugate is promising as an anti-inflammatory diagnostic and therapy agent and deserves further investigation.

### 5.2. Nitroxyl Radicals

Stable nitroxyl radicals demonstrate high biological activities due to their ability to diminish the effects of oxidative stress. This is, in particular, due to their reaction with peroxyl radicals, in which nitroxyls act as catalytic radical-trapping antioxidants [234]. The paramagnetic properties of nitroxyl radicals permit using them also as contrast agents for MRI. Therefore, nitroxyl radicals, being less toxic than gadolinium and manganese complexes, can act as both CM for MRI and antioxidants [235]. A significant biomedical application of nitroxyl radicals is imaging of a redox status (and redox imbalance) in different tissues, which is important, for example, in cancer diagnostics and treatment [235].

Nitroxyl–drug conjugates can be used for various theranostic applications. In particular, a nitroxyl-labeled analogue of the conventional anticancer drug Lomustine was synthesized and used as a low toxic spin label for noninvasive MRI of blood–brain barrier permeability and a drug for cancer therapeutics [236].

To increase the intensity of MRI signals, multispin molecular systems are synthesized (with several nitroxyl radical residues in one molecule) [237]. For theranostic purposes, heparin–polynitroxyl structures were synthesized in which a number of nitroxyl residues were bound to a heparin macromolecule; their antioxidant and magnetic (electron paramagnetic resonance (EPR) and MR) properties were studied [238]. Chitosan–polynitroxyl systems were synthesized and studied as well [239].

### 5.3. Theranostic Antioxidant Nanomaterials

At present, nanomaterials are becoming more and more widespread in various biomedical applications (see, e.g., the recent review [240]). A small group of nanomaterials that can be used as theranostic agents (more detailed consideration was beyond the scope of this review) is considered below.

#### 5.3.1. Cerium Oxide Nanoparticles

Cerium oxide nanoparticles have attracted interest for their regenerative, multi-enzymatic scavenging of ROS, possessing unique antioxidant/catalytic properties [241,242,243]. For theranostic purposes, cerium oxide nanoparticles with fractions of Gd (up to 50%) have been prepared. In these structures, Gd was incorporated into the crystal structure of cerium oxide nanoparticles. Such nanoparticles have both properties of a contrast agent in MRI investigations (due to Gd content) and antioxidant properties (due to Ce) [244]. The nanoparticles exhibited high *T*_1_ relaxivity and the potential to act as scavengers of ROS. The presence of Ce^3+^ sites and oxygen vacancies at the surface plays a crucial role in providing the antioxidant properties [245]. Such nanoparticles, coated with dextran, demonstrated dose-dependent selective cytotoxicity to cancer cells [246] and have a potential for future theranostic applications.

Cerium oxide nanoparticles (as such or doped/functionalized) are extensively used in different theranostic systems [247].

#### 5.3.2. Iron Oxide Nanoparticles

Iron oxide (Fe_3_O_4_, magnetite) nanoparticles possess magnetic properties and can be used as contrast agents in MRI [248]. The ease of functionalization of their surfaces with different types of biomolecules (antibodies, peptides, sugars, etc.) creates the possibility of using them for various theranostic applications [249,250]. Magnetite nanoparticles functionalized with quercetin exhibit antioxidant, anti-inflammatory and antimicrobial activities [251]. Magnetite nanoparticles were also functionalized with poly(ethylene glycol) + D-glucosamine to specifically target breast cancer cells. The nanoparticles exhibited very slight cytotoxicity on normal human kidney cells [252].

Therefore, functionalized iron oxide nanoparticles have a high potential for the development of new theranostic agents. The properties and biomedical applications of magnetic nanoparticles are summarized in recent reviews [253,254].

### 5.4. Summary

Table 2 summarizes the types of hybrid contrast/antioxidant media having a potential for theranostics.

## 6. Discussion

Considering the large amount of research and clinical data on the use of antioxidants for the prevention or reduction in contrast-induced injuries (mostly CIN), one can notice the controversial character of some of these data, which is emphasized in a number of reviews and meta-analyses (see, e.g., [11,19]). For example, although NAC began to be used as a remedy against CIN more than 20 years ago, the different meta-analyses of clinical applications of NAC against CIN exhibited controversial results (see Table 1). The same situation is observed for vitamins C and E and statins (Table 1). In spite of the high antioxidant activity of glutathione, the use of GSH for the prevention of CIN in elderly patients did not yield distinct (positive) results [79,80] (see also Table 1). Some researchers do not recommend the use of antioxidants to prevent CIN but recommend the use of hydration instead [17]. This may be due to some shortcomings in the statistical processing of the results. But more likely, it could be the complex nature of the diseases in patients (especially elderly), who, along with the main disease, often have a whole gamut of other diseases. Therefore, the improvement of CIN after administration of an antioxidant drug may not be seen against the background of other diseases. In addition, for an effective action against CIN, a drug, along with an antioxidant, should also have a range of other properties: anti-inflammatory, antiapoptotic and immunomodulatory; this may not always be the case. Furthermore, the mode of administration (oral or intravenous), doses, timing (before taking CM or with CM) and frequency of drug administration (single or multiple) are also important for the effective action of the drug. All these factors should be considered and require comprehensive research for the efficient use of known and the development of new drugs against CIN.

Along with the external administration of antioxidants (both exogenous and endogenous), which does not always yield the expected effect, it is important to pay more attention to the stimulation (induction) of internal endogenous antioxidants in the patient with CIN. Such endogenous antioxidants, in particular, include bilirubin. It has been noticed that higher serum bilirubin levels corresponded to a lower risk of CIN and fewer cardiovascular complications [124,125]. As considered before, the “heme–biliverdin–bilirubin” series involving the enzyme HO-1 can serve as an effective antioxidant system protecting from CIN. Hence, stimulation of bilirubin levels by induction of HO-1 may lead to reducing CIN. Since there are a large number of HO-1 inducers (including naturally derived ones) [141,142], induction of HO-1 may be a promising method for combating CIN and deserves detailed study. However, it is necessary to consider that very increased HO-1 expression may lead to the development of serious diseases [143] and that total serum bilirubin at high levels is toxic [144].

Apart from the use of individual antioxidants against CIN, it is promising to create universal systems that combine antioxidant and contrast properties, which can be used both for the diagnosis of diseases and for their therapy (theranostics). Especially promising is the use of nanomaterials, which are now becoming more widespread. In particular, recently created novel CM based on DND particles with grafted paramagnetic metal cations [31,32,33,34], the use of cerium oxide nanoparticles doped with Gd [244,245,246] or the creation of magnetic nanoparticles (magnetite and others) functionalized with antioxidant ligands [249,250,251,252] for the purposes of MRI and therapy can give a new impetus to the development of novel contrast and theranostic systems.

## 7. Conclusions

Experimental and clinical studies have shown that ROS play a crucial role in the pathogenesis of CIN. The controversial character of some of the research and clinical data on the use of antioxidants for the prevention or reduction of CIN could be due to the complex nature of the diseases in patients, who usually experience other diseases along with the main disease. Furthermore, a drug effective against CIN, along with antioxidant ability, should also have other important properties: anti-inflammatory, antiapoptotic and immunomodulatory. All these factors, together with some others, should be considered for the effective use of known and the development of new drugs against CIN.

Promising is the stimulation of internal endogenous antioxidant systems in patients with CIN, in particular, the “heme–biliverdin–bilirubin” series involving the enzyme HO-1, which may be a potent method for combating CIN and needs further research.

Also promising is the creation of universal systems that combine antioxidant and contrast properties which can be used in theranostics. Especially potent is the use of nanomaterials as contrast and theranostic agents. In particular, recently created novel CM based on DND particles with grafted paramagnetic cations, the use of cerium oxide nanoparticles doped with Gd or the creation of magnetic nanoparticles functionalized with antioxidant ligands can provide a new stimulus to the development of novel contrast and theranostic systems.

## Figures and Tables

**Table 1:** Qualitative results of clinical trials and research studies of antioxidant remedies for prevention and attenuation of CIN.

Antioxidant	Results of Using against CIN	Clinical Trials (Studies)
NAC	positive	on humans [53,54,55,56,57,58,60], meta-analyses [59,61,71]
	no results	on humans [62,63,64,65,66,67], meta-analyses [68,69,72]
Glutathione	no results	on humans [78,79,80]
Vitamin C	positive	on humans [96,97,98], meta-analyses [94,95]
	no results	on humans [99,100,101,102,103,104]
Vitamin E	positive	on humans, meta-analyses [113,114]
	no results	on humans [115,116]
Bilirubin/HO-1	positive (expected)	Association of bilirubin level with CIN was found in [124,125,139]; induction of HO-1 (on animals) [140]
Melatonin	positive	on animal models [146,149,152,153,154,155]
L-carnitine	positive	on humans [162,163,164,165]
Statins	positive	on humans [170,171,172,173], meta-analyses [174,175,176,177], on animals [178,179]
	controversial	on humans [180]
Probucol	positive	on humans [181,182], meta-analyses [183,185]
	no results	on humans, meta-analysis [184]
MESNA	positive	on humans [190,192]
Resveratrol	positive	on animal models [195,196], in vitro [197,198,199,200]
Astaxanthin	positive	on animal models [204,205,206,207]
Lycopene	positive	on animal models [200,209,210,211]
Green tea extract	positive	on animal models [219,220]
GSPE	positive	on animal models [224,225]
Curcumin	positive	on animal models [228,229]
Xylose-pyrogallol conjugate	positive	on animal models [231]

**Table 2:** Types of hybrid contrast/antioxidant media having a potential for theranostics and their studies.

Hybrid Medium	Studies
Gd complex/RosA conjugate	in vitro and on animal model (antioxidant and MRI) [233]
Nitroxyl radicals	MRI, EPR, redox properties [235,237]
Nitroxyl–Lomustine (anticancer)	MRI, blood–brain barrier permeability [236]
Nitroxyl radicals–heparin	in vitro and on animal model (antioxidant and MR properties) [238]
Nitroxyl radicals–chitosan	in vitro (antioxidant properties, EPR) [239]
Cerium oxide nanoparticles doped with Gd	dynamic light scattering, Zeta potential measurement, X-ray diffraction, high-resolution transmission electron microscopy, near edge X-ray absorption fine structure, MRI properties, antioxidant properties in vitro [244,245] For the nanoparticles coated with dextran—cancer cytotoxicity [246]
Functionalized by quercetin magnetite nanoparticles	antioxidant and antibacterial properties in vitro, FTIR, Raman, TEM, X-ray spectroscopies, magnetic properties [251]
Functionalized by PEG + D(+) glucosamine magnetite nanoparticles	XRD, VSM, FESEM, and FTIR analyses; MRI properties, particle size, zeta potential, biodistribution analysis, very slight kidney cytotoxicity [252]

## Data Availability

Data sharing not applicable.
